# Evidence of Skin Barrier Damage by Cyclic Siloxanes (Silicones)—Using Digital Holographic Microscopy

**DOI:** 10.3390/ijms21176375

**Published:** 2020-09-02

**Authors:** Krystyna Mojsiewicz-Pieńkowska, Ewa Stachowska, Dominika Krenczkowska, Dagmara Bazar, Frans Meijer

**Affiliations:** 1 Department of Physical Chemistry, Faculty of Pharmacy, Medical University of Gdańsk, al. gen. Józefa Hallera 107, 80-416 Gdańsk, Poland; d.krenczkowska@gumed.edu.pl (D.K.); dagmara-b@gumed.edu.pl (D.B.); 2 Department of Metrology and Measurement Systems, Faculty of Mechanical Engineering and Management, Poznan University of Technology, ul. Piotrowo 3, 60-965 Poznan, Poland; ewa.stachowska@put.poznan.pl (E.S.); frans.meijer@os.put.poznan.pl (F.M.)

**Keywords:** stratum corneum barrier organisation, skin barrier, disrupted skin barrier, siloxanes–skin lipids interaction, skin lipids interaction, clusters and canyons, lacunae, intercluster region, skin penetration, penetration pathways, digital holographic microscopy, DHM, cyclic siloxanes, silicones

## Abstract

Cyclic siloxanes (D4, D5, D6) are widely used in skin products. They improve skin sensory properties and alleviate dry skin, but there is still one report (published 2019), which regards their effects on the destruction of the skin barrier, by using fluorescence microscopy and attenuated total reflection Fourier-transform infrared spectroscopy (ATR-FTIR). A new skin-imaging technique, digital holographic microscopy (DHM), was used for the first time to investigate the impact of D4, D5, and D6 on the skin barrier. We observed irreversible damage of the stratum corneum due to the interaction with cyclic siloxanes. These substances changed: (a) the first level of the skin barrier through destabilization of the intercellular lipid lamellae and destruction of the corneocyte structure (measured with axial nanometer resolution), (b) the second level by collapse of not only corneocytes but also of a significant part of the clusters, leading to the loss of the stratum corneum integrity and formation of the lacunae, (c) the third level as an effect of the change in the surface geometrical topography of the stratum corneum and disruption of the integrity of this skin layer, measured with lateral micrometer resolution. DHM allowed also to identify an important pathway for substances to penetrate into the skin through canyons surrounding the clusters. Our investigations provide advanced information for understanding the mechanisms by which various substances pass the skin barrier, including uncontrolled diffusion into the skin.

## 1. Introduction

It is known that the human skin is composed of characteristic layers and the stratum corneum (SC) is particularly crucial for the skin barrier and responsible for protection against various factors entering the skin (e.g., xenobiotics, allergens, microorganisms). It was proved that some substances (topical excipients or formulation components) present in pharmaceutical skin products, personal care products, and cosmetics (e.g., surfactants, plant-derived oils, paraffin, petrolatum, liposomes, emollients) can cause changes in the SC structure and damage the skin barrier, which thereafter may cause medical and toxicological effects [1,2,3,4,5,6,7,8,9].

Some of the most popular substances widely used in skin products are cyclic and linear siloxanes, commonly named silicones. With their unique chemical and physical properties, siloxanes provide a wide array of benefits in skin care products (e.g., sensory and texture enhancement, emollience and high spreadability, with a transient to long-lasting effect, resistance to washing off, occlusive or water vapor-permeable properties, protection, cleaning) [10].

Siloxanes improve skin sensory properties and alleviate dry skin, but there is still one report (published 2019), regarding their effects on skin barrier properties, including interaction with lipids and proteins of the SC, proved by using fluorescence microscopy and attenuated total reflection Fourier-transform infrared spectroscopy (ATR-FTIR) [11]. To study this in more detail, we selected the cyclic siloxanes D4, D5, and D6, based upon the current state of knowledge about these compounds; especially with respect to: (a) widespread use in medical products for skin, cosmetics and personal care products not only intended for adults (e.g., Zeraderm Rofil Medical, La Roche-Posay Hydreane Riche cream, Vichy Aqualia Thermal cream, Garnier Regenerating body lotion) and children (e.g., Aderma Epitheliale Pierre Fabre Cosmetique), but also for infants (e.g., Penaten cream, Emolium) and (b) significant environmental pollution (in particular water, sediment, soil, air, and dust), increasing the risk of human exposure to these compounds, especially on exposure through the skin, posing even toxicological problems [11,12,13,14,15].

So far, only two studies have been published, by our research team, which proved that the human skin provides no barrier for D4, D5, and D6 [11,16]. We also proved that the compounds analyzed can bio-accumulate, particularly in the SC, but also in the epidermis and the dermis. Furthermore, identification of cyclic siloxanes in receptor fluids indicated an increased risk of absorption into the organism via the blood and lymphatic vessels located in the dermis [11,16].

In this study, we focus on the impact of different cyclic siloxanes on the SC. We analyzed skin surface geometrical topography and the structural changes in clusters, canyons (intercluster region), corneocytes, and intercellular lipid lamellae, which constitute the basis of the skin layer structure. Digital holographic microscopy (DHM) was used for the first time to examine human skin—structure, barrier function, penetration, penetration pathways, and interaction with xenobiotics. It should be emphasized that using DHM contributed to the understanding of several important aspects. The presence and role of the SC multilevel structure in the skin barrier function has not been described in the literature in this way so far. In scientific literature, the skin barrier is described principally in relation to the characteristic structure of corneocytes and the surrounding lipid matrix. In this work, we propose a multilevel structure of the SC as an innovative way of describing the skin barrier. We consider the skin barrier in the context of corneocytes and matrix only as a simplification of this complex structure. We furthermore explain the advanced mechanism of losing the skin barrier due to the interaction between substances and the structures of the SC. This knowledge can help to protect the human skin against undesirable side effects of various substances (including some siloxanes) used in skin products. DHM has also already been used to study human tissues, blood cells, and the pathophysiology of live biological cells, including nerve cells [17,18,19,20,21,22,23,24], however this microscopic technique has not yet been used in skin studies.

## 2. Results and Discussion

**Figure 1:**
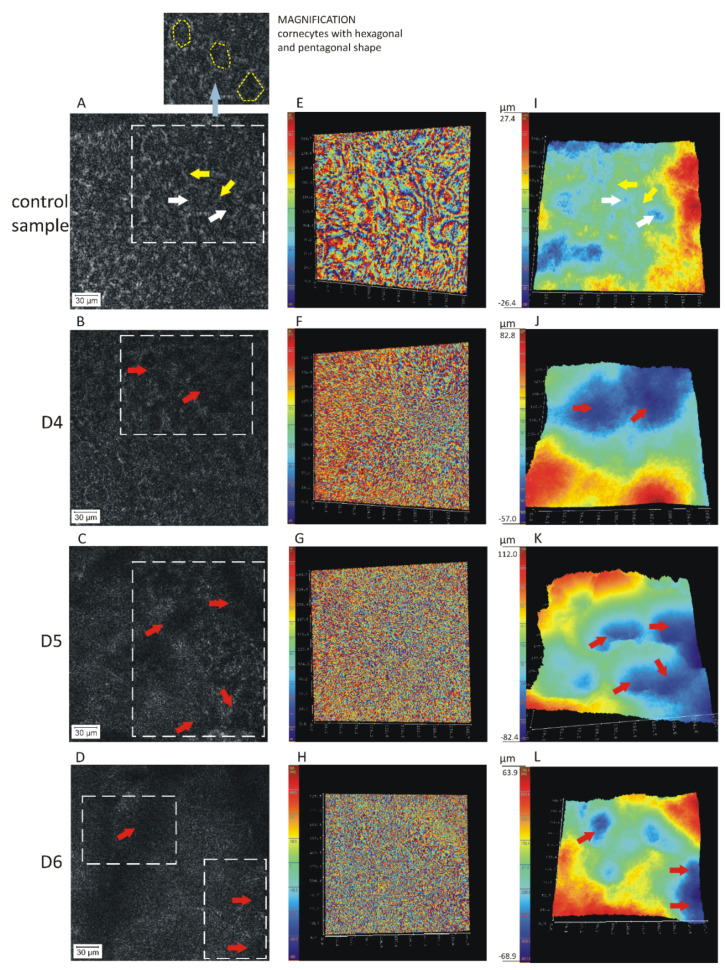
Intensity images (**A**–**D**), wrapped phase images (**E**–**H**), and unwrapped 3D phase images (**I**–**L**) of changes in the human stratum corneum structure under the influence of cyclic siloxanes (BFJ;CGK;DHL) compared with the control sample (**A**,**E**,**I**); at 20× magnification. The numbers at images (**I**–**L**) are determined with reference to the mean value of the respective image.

**Figure 2:**
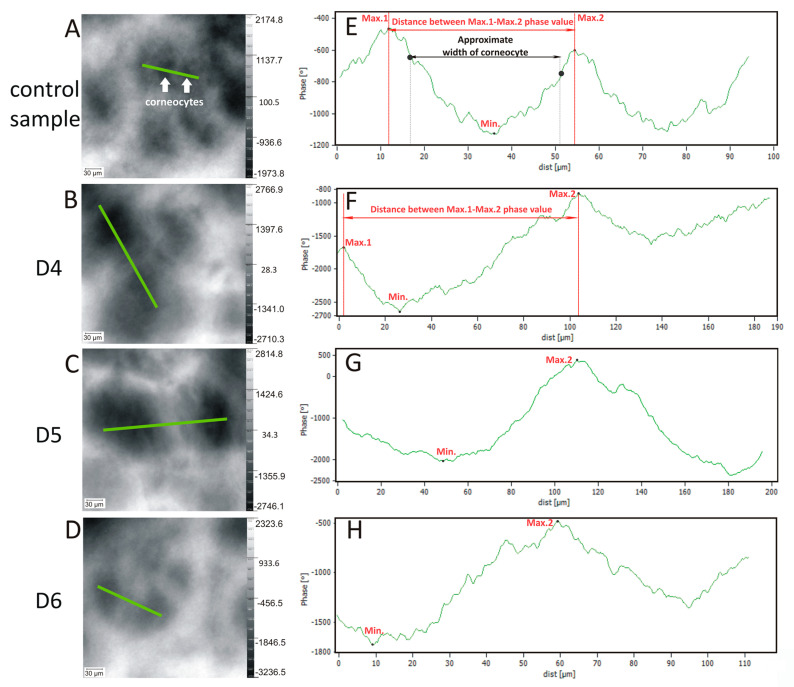
Holographic images (unwrapped phase image) and corresponding profile lines of the differences in phase along the green lines in (**A**–**D**), when the human stratum corneum structure is changed under the influence of cyclic siloxanes compared with the control sample, where the control sample (**E**) and test samples (**F**–**H**); the magnification 20×. The values of the phase are determined with reference to the mean value in the respective image.

**Figure 3:**
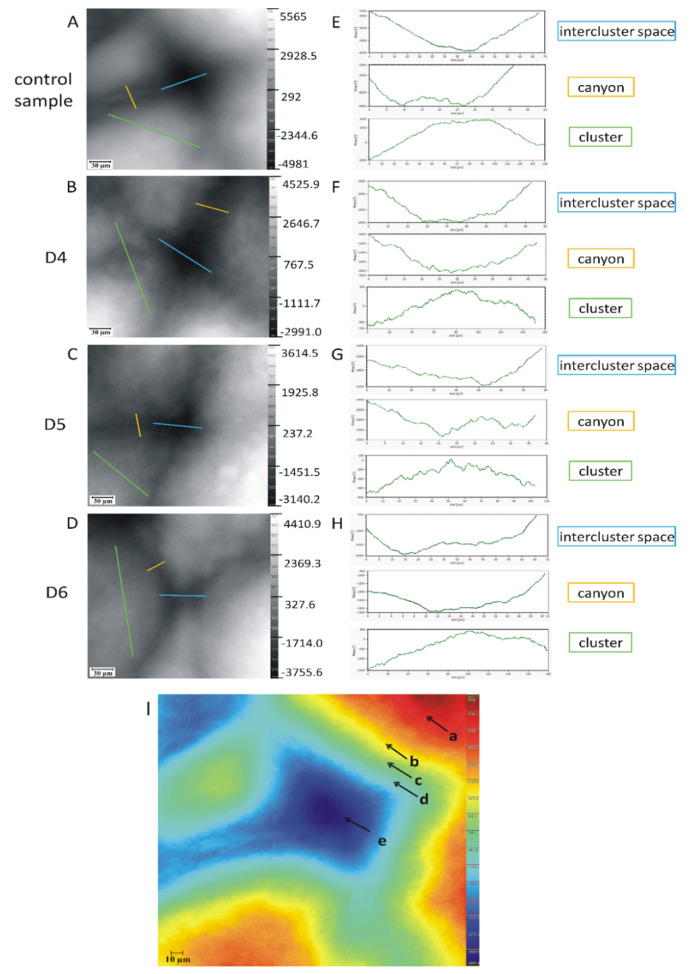
Comparison of unwrapped phase images of clusters and canyons in the stratum corneum for test samples (**B**–**D**) and a control sample (**A**) with corresponding profile lines; 20× magnification (**E**–**H**). The spaces between the clusters are marked in blue, the clusters in green and the canyons in yellow; a representative unwrapped phase image of clusters (**I**), canyons and intercluster space in the *SC*: a (red color)—clusters as an agglomeration of corneocytes, the distance along the cluster consisting of 15–30 corneocytes (parallel to the surface of the skin) varies between 100–250 µm; b (yellow color)—cluster boundaries—part of the cells are located on the cluster boundaries; c–d (green and light blue color)—lipid layers of canyons with hydrophobic and lipophilic properties, characterized by low water content and less resistance to penetration than the average intercorneal space; e (dark blue color)—funnel structure of intercluster space—resembles an inverted, flat arc with a peak, reaching up to 5–10 µm to the epidermis; characterized by low water content and less resistance to penetration, the deepest point reaches the dense network of blood and lymphatic vessels in the dermis. The values of the phase are determined with reference to the mean value in the respective image.

**Figure 4:**
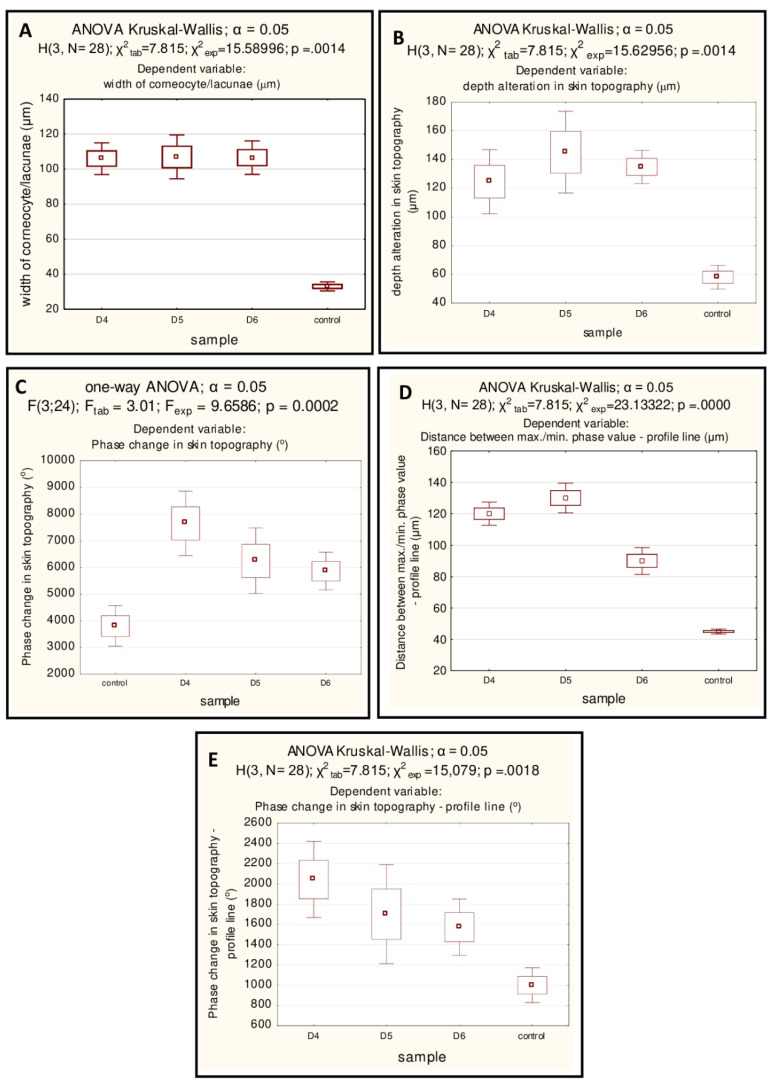
Statistical data evaluation reflecting the stratum corneum geometrical topography and structural changes. Comparison the quantitative results after of siloxanes application with control sample: (**A**)—the average widths of the corneocytes and lacunae [um], (**B**)—depth alteration in skin geometrical topography, (**C**)—phase differences (°), (**D**)—phase change (°)—profile line, (**E**)—distance between max./min. phase value (μm).

The comparative studies between test samples (where we applied D4, D5, or D6 siloxane to isolated epidermis containing the SC structure) and control samples (without application of siloxane) were conducted. It should be pointed out that due to the low power of the laser radiation (200 μW/cm^2^), our samples were not changed due to e.g., photodegradation. Holographic microscopy revealed the stratum corneum geometrical topography and structural changes after application of cyclic siloxanes on the skin (Figure 1, Figure 2 and Figure 3, Table 1). We observed changes in the surface topography of the stratum corneum as well as lacunae formation in the intercellular space. We also performed statistical tests on the results (Figure 4). The consequence of impact of cyclic siloxanes on the skin barrier is presented in Figure 1 which shows the following images: intensity, wrapped phase, and unwrapped phase.

We found on the basis of the intensity images that the control samples (Figure 1A, magnification 20×, size 260.7 × 260.7 µm) show corneocytes with a characteristic hexagonal or pentagonal shape with a diameter of about 33 μm (white arrow) surrounded by the extracellular lipid matrix (yellow arrow). Different results were observed in the case of the test samples (Figure 1B–D) showing a disturbed or even destroyed structure of the SC (red arrow). As a result of the application of cyclic siloxanes (Figure 1B–D,F–H), the SC surface no longer has visible characteristic structures: corneocytes and lipid matrix, contrary to the control samples (Figure 1A,E). In some areas, lacunae (gaps) formation in the intercellular space was also observed as a result of the interaction with D4, D5, and D6 (Figure 1B–D,J–L). A similar effect was also noticed by other researchers, who studied the interaction with mineral-derived (paraffin and petrolatum) and plant-derived (almond and jojoba) oils using confocal Raman microscopy [1]. These lacunae point to the fact that the regular organization of the SC has been damaged in those places because part of the lipids from the intercellular space was extracted by lipophilic cyclic siloxanes. Irreversible disorder of the SC structure due to lacunae (open space) formation can be explained by permeation of various cyclic siloxanes (molecular weight 297–445 Da and logP = 5.1–8.87) through the stratum corneum, epidermis and dermis up to the receptor fluid. This has been proved in previous studies by our research team [11,16].

Based on the unwrapped 3D phase images (Figure 1I–L), the average width of the corneocytes was found to be 33 µm (RSD = 11%) (Figure 1I, white arrows), the average lacunae after D4 application 106 µm (RSD = 12%) (Figure 1J, red arrows), the average lacunae after D5 application 107 µm (RSD = 16%) (Figure 1K, red arrows), and the average lacunae after D6 application 107 µm (RSD = 12%) (Figure 1L, red arrows). In each case, the average values were determined from seven independent measurements, which are presented in Table 1 and Figure 4A in statistical data evaluation. It should be emphasized that unwrapped 3D images provide very important information about changes in the lateral (horizontal) plane of the SC structure, as well in the axial (vertical) direction, due to effects of the application of cyclic siloxanes. These compounds perturbed the SC lipid bilayers and induced lacunae formation in the intercellular space. Measurements in the axial direction were performed with nanometric resolving power. The results for the axial direction can be understood as a combination of geometrical topography and differences of refraction index for different skin structure [25]. The relative changes in the axial direction were much smaller for the control samples (about 58 μm (RSD = 19%), see Figure 1I) than for the test samples. The latter were about 125 μm (RSD = 23%), 145 μm (RSD = 25%), and 134 μm (RSD = 11%) for D4, D5, and D6 respectively (Figure 1J–L). The biggest lacunae formations were observed for D5. We can conclude that all cyclic siloxanes change the topography of the surface of the SC. The surface topography of the control sample is much more flat and compact. These data as the depth alteration in skin topography (μm) are presented in Table 1 and also Figure 4B in statistical data evaluation.

The loss of the regular *SC* structure in the test samples can be also deduced by comparing the wrapped image 1E with the images 1F–H. The distances between the interference fringes are smaller for the test samples (Figure 1F–H) than for the control sample (Figure 1E). This indicates that their greater density is due to greater surface variation (topography).

Observation of the degree of the structural change of the *SC* is also possible using the unwrapped images with a vertical scale of the phase change of the wavefront in degrees after passing the sample. Figure 2 shows examples.

Clusters of corneocytes, the areas marked with white arrows in Figure 2A, are clearly visible, surrounded by the lipid matrix. The width of the corneocytes was approximately 35 μm. In the test samples the structure was destroyed, which contributed to the lacunae formation in the intercellular space (Figure 2B–D), already mentioned above. The vertical scale on the right side of the images shows the size of the phase change in the axial (vertical) direction (phase change in skin topography [◦]) allowing a quantitative assessment of the destruction of structures in this layer of the skin. For the test samples, the average depth differences, expressed as the change in the phase, were assessed on the basis of seven independent skin samples. These values were as follows: 7657° (RSD = 21%) for D4, 6252° (RSD = 26%) for D5, and 5868° (RSD = 16%) for D6, and for the control sample only 3810° (RSD = 27%). These data as the phase change in skin topography (°) are presented in Table 1 and also Figure 4C in the statistical data evaluation.

Examples of linear profiles (along the green lines in Figure 2A–D) were also registered and are shown in Figure 2E–H. The distance marked along the x axis in Figure 2E reflects approximately the width of a corneocyte; here 35 μm. For all samples, the distance between the points Max.1 and Max.2 phase value [μm] was determined. Final results were obtained from measurements of seven samples and presented in Table 1 and also Figure 4D. For the control sample, the average distance between neighboring points Max.1 and Max.2 was 45 μm with RSD = 5%. Changes in the corneocytes were identified for the test samples as the lacunae in intercellular space with an average width for D4 of 120 μm (RSD = 8%), for D5 of 130 μm (RSD = 10%), and for D6 of 90 μm (RSD = 13%). The vertical distances between the points Max.2 and Min. (phase change) were for: control sample 1000° (RSD = 24%), D4 2043° (23%), D5 1700° (RSD = 25%), and D6 1571° (RSD = 24%). Final results were obtained from the measurements of seven samples and presented in Table 1 and also Figure 4E in the statistical data evaluation. The results show the phase difference which depends on the presence of structures with different refractive index or thickness. The results obtained confirm that the changes occurred as a result of disorder of the coherence of the organized stratum corneum under the influence of cyclic siloxanes.

The crucial achievement of this research was the identification of the presence of clusters and canyons (detailed description in Table 2), which proves the existence of a more organized structure of the stratum corneum (Figure 3). Clusters are an agglomeration of corneocytes, while the canyons represent the lipid spaces located between clusters, reaching even the dermo-epidermal junction [26]. It should be emphasized that canyons have been so far only described in a few scientific manuscripts. They were indicated as an important, alternative pathway of diffusion of substances into the skin, including a transport route for dermal formulations in the form of nanoparticles [26,27,28,29,30]. Some studies have shown that canyons are characterized by limited resistance to penetration of xenobiotics applied to the skin [1,26,27,28,31,32,33].

In the images in Figure 3A–D, the spaces between the clusters are marked in blue, the clusters in green, and the canyons in yellow. Linear profiles enabled the measurement of the size of these structures. Preliminary studies have shown that the size of the clusters vary in a range of 100–180 μm depending on the number of corneocytes in an agglomeration. The widths of the canyons are approx. 25 μm, while the spaces between the clusters are in the range of 65–80 μm. Influence of the cyclic siloxanes on the skin surface is also visible. For the control sample, the surface was uniform in contrast to the test samples where shadows are visible (Figure 3B–D). Noteworthy is also the fact that in the case of the test samples, all line profiles for canyons, clusters, and intercluster spaces (Figure 3F–H) are jagged compared with the control sample (Figure 3E), which is evidence of a change in the surface geometrical topography of the SC under impact of cyclic siloxanes.

The identification of more organized structure of the SC (clusters and canyons) was an important observation. In Figure 3I, the agglomeration of corneocytes forming clusters is clearly visible (part a, red). They are surrounded by lipids. (part b, yellow). The changing intensity shown as a color range from yellow (part b), through green (part c), light blue (part d), to dark blue (part e), which indicates the existence of canyons. Figure 3I shows also the characteristic structure of the funnel-shaped canyons and the lipid layer. They tightly surround the agglomeration of the corneocyte-forming clusters. This level of SC organization indicates additional integrity of this skin layer, which affects additional reinforcement. It should be pointed out that in the literature, the description of skin barrier often regards only the explanation of the regular arrangement of several layers of corneocytes, tightly surrounded by the lipid matrix. However, this does not reflect the complex structure of the stratum corneum. This, in turn, increases the skin barrier. The canyon may be an important, additional way of transporting substances into further layers of the human skin. The centrally located space between the clusters (Figure 3I) confirms the existence of a specific structure of funnel-shaped canyons, where the largest depth is pointed to by 3Ie (part dark blue).

### Statistical Data Evaluation

Statistical evaluation of the results included the analysis of five parameters described below (A–E). For each sample group (control and test samples after application of D4, D5, D6), *n* = 7 independent measurements of presented parameters were performed. The statistical distributions of the (A) width of the corneocytes and lacunae, as well as (B) the phase differences reflecting the skin topography changes (Figure 1I–L, Table 1), were normal (Shapiro–Wilk test), but the data did not show homogeneity of variance (Levene’s or Brown–Forsythe test). We used a non-parametric one-way ANOVA Dunn’s post hoc test to verify whether average values from different measurements are equal. We found that the (A) width of corneocytes and lacunae as well as the (B) phase differences differed statistically significantly for all test samples (treated with D4, D5, and D6 silicones) in comparison with the control sample, while between the test samples, no significant difference was found. Results given in Figure 4A,B confirm observed destabilization of the intercellular lipid lamellae and formation of gaps as a result of occurring interactions.

In the case of (C), the phase change in the skin topography (see Figure 2A–D and Figure 4C, Table 1), associated with assessment of the structures destruction in the vertical direction, all data met the assumptions of normal distribution (Shapiro–Wilk test) and homogeneity of variances (Hartley’s test); we performed therefore, a one-way analysis of variance (ANOVA). We observed the existence of differences in the averages between groups analyzed. The same conclusions as described above (presented on the Figure 4B) could be drawn from the Tukey’s post hoc analysis. In the case of the analysis of (D), the distance between maximum and minimum phase difference (max.-min. see Figure 2E–H), connected with the disruption of the layer integrity observed in lateral direction, the non-parametric counterpart of ANOVA was used, as data met the assumption of the variances homogenity (Levene’s and Brown–Forsythe tests) but did not show normal distribution (Shapiro–Wilk test). In order to further evaluation of the obtained by Kruskal–Wallis test differences, Dunn’s post hoc test was used. Compared with the control sample, only the test samples D4 and D5 differed significantly (Figure 4D) (E). Phase differences from profile lines (vertical direction resulting from silicones changes) did not meet the assumption of normal distribution (Shapiro–Wilk test), while the condition of homogeneity of variances was met (Levene’s and Brown–Forsythe tests). Differences between the groups of test and control samples were found by using the Kruskal–Wallis test (Figure 4E). Statistically significant differences were observed only between the test sample D4 compared with the control ones (Dunn’s post hoc analysis), although disorder of the structures in vertical axis is also noticeable in case of the rest of analyzed siloxanes.

## 3. Methods

### 3.1. Test Substances

The following cyclic siloxanes were used as test substances: octamethylcyclotetrasiloxane (D4); molecular weight 297 Da, LogP = 5.10, decamethylcyclopentasiloxane (D5); molecular weight 371 Da, LogP = 8.06, dodecamethylcyclohexasiloxane (D6); molecular weight 445 Da, LogP = 8.87. They were obtained from Sigma-Aldrich, Steinheim, Germany.

### 3.2. Research Methodology

We developed our research methodology, including the ex vivo skin sample preparation, based on the official guidelines for the study of dermal absorption of xenobiotics, published by the Organization for Economic Cooperation and Development (OECD) and the World Health Organization (WHO) [11,27,34,35,36].

### 3.3. Preparation of Ex Vivo Skin Samples

The approval to use human cadaver skin for our experiments was approved by the Independent Bioethics Commission for Research at the Medical University of Gdańsk (no NKBBN/309/2013; 8 July 2013). We confirm that all experiments were approved by the Independent Bioethics Commission for Research at the Medical University of Gdańsk and all experiments were performed in accordance with relevant guidelines and regulations. The skin samples from human cadaver skin were obtained from the Department of Forensic Medicine, Faculty of Medicine, Medical University of Gdańsk which received the informed consent was obtained from a LAR (legally authorized representative). These samples, with dimensions: 15–20 cm length and 2–3 cm width, were obtained from the abdomen region and came from 3 males and 3 females at the age of 35–50 years. The average age was 40 years for the females and 45 years for the males. The samples were collected within 48 h after death. They were prepared according to the method of Krenczkowska et al. [11] and kept frozen until analysis. Skin pieces with an area of ~1 cm^2^ were after defrosting checked for integrity using a magnifier with a 4-fold magnification as well as using an electrical resistance technique (ER). All skin sample measurements showed a value of more than 2 kU/cm^2^ which indicated skin integrity [37,38].

### 3.4. Preparation of the Samples to Microscopic Investigation

The epidermis was obtained using the heat separation technique (temperature 60 °C, time 30 s). Pieces of epidermis (~1 cm^2^) were subsequently placed on microscopic slides with the SC upwards. In the case of the control samples (no application of cyclic siloxanes), the first three layers of the human skin epidermis were removed by a tape-stripping technique. The surface of a control epidermis was observed directly after preparation. In the case of the test samples, siloxane (D4, D5, or D6) was directly applied on the epidermis surface in an amount that allowed to cover the whole surface. The test samples prepared were then incubated in a closed Petri dish (with an insulating parafilm) for 1 h at 32 °C. Thereafter the excess amount of test silicone was removed and the upper skin surface was dried with absorbent paper. Similarly to the control samples, the first three layers of the test samples were removed by the tape-stripping technique.

### 3.5. Digital Holographic Microscopy

**Figure 5:**
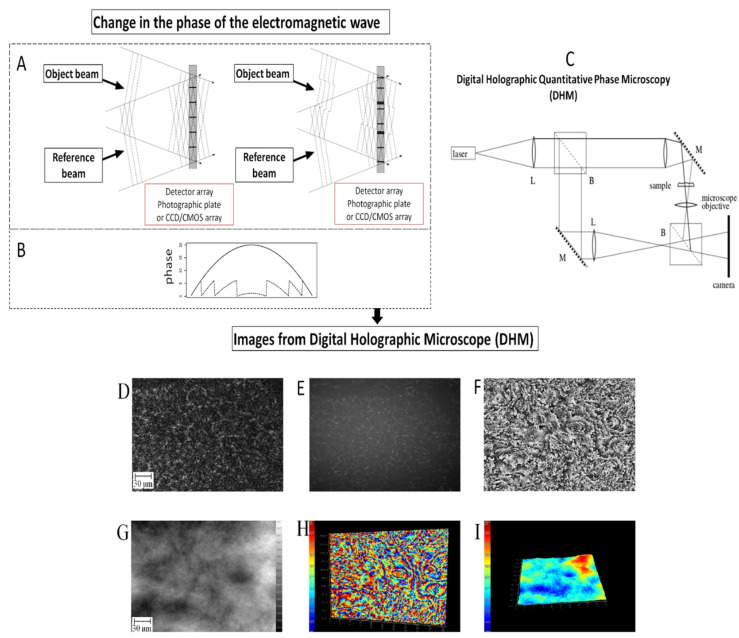
Digital holographic microscopy. (**A**)—left: the interference pattern from a plane reference beam and a plane object beam; right: when part of the wave-front of the object beam is shifted due to depth differences in the object, the interference pattern is changed: depth distances are encoded in the hologram registered by the detector; (**B**)—the dashed line is the wrapped phase along a line across a sample; the full line is the result of unwrapping and is a measure of the optical thickness along this line; (**C**)—optical scheme of a digital holographic transmission microscope; L = lens, B = beam-splitter, M = mirror; (**D**)—an intensity image, as in classical optical microscope with monochromatic illumination; (**E**)—hologram; (**F**)—wrapped phase image; (**G**)—unwrapped phase image; (**H**)—3D representation of (**F**); (**I**)—3D representation of (**G**).

An optical wave can be described by the following equation: y = A e^i(φ−ωt)^. No detector can follow the very high frequency ω, so we can only detect an average in time, so we simply drop the time-dependent part. When we make a classical image we register only the intensity over the cross-section of the beam (wave-front) hitting the detector, photoelectric, photographic or our eye. This wave-front can be given as A(i,j)e^iφ(i,j)^, where i and j are the coordinates on the surface of the detector, A(i,j) the amplitude, and φ(i,j) = 2π δ(i,j)/λ the phase, with λ the wavelength of the light used and δ(i,j) the difference in path length of a point (i,j) of the object compared with a given reference point. But we can only measure the intensity I = AA *. When we register an image of our object then the intensity on each point of the detector is a measure of how much light is reflected (or emitted) from each point of our object. But if we want to measure depth differences in our object, we have to register the phase too. Holography is a technique which allows us to register both amplitude and phase [39]. When we illuminate our object with a laser and combine the reflected light with a reference beam, coherent with the object beam (see Figure 5A), this results in an interference pattern. When the object is flat (and the reference beam is a simple parallel beam, as in Figure 5A left) both beams have a flat wave-front and the resulting interference pattern is simple.

But when the object shows depth differences the wave-front of the object beam is no longer flat, some parts are delayed (as in Figure 5A right) and some of the maxima of the interference pattern are shifted. Thus both the amplitude and the phase are encoded in the interference pattern: we have made a hologram. When we now illuminate a classical (photographic) hologram with the reference beam only, then the wave emerging from the hologram, resulting from the multiplication of the reference beam with the hologram, contains also a beam equal to the beam from the object alone. When we look into this beam we “see” the original object. On the retina of our eye, we get the same wave-front as from the object alone. Holography is simply wave-front reconstruction [40]. When using a photoelectric detector array (CMOS or CCD) we can however not look through the detector and the information is stored as an array somewhere in a computer memory. If the reference beam is simple (flat or spherical) we can the phase of this beam easily describe as an array with a simple formula. Multiplying the array containing our hologram with this phase array gives us the wave-front originating from the object and taking the square of this wave-front gives us the intensity. When a wave-front in one plane of an optical beam is given, we can easily calculate the wave-front in another plane [41]. In this way, we can reconstruct the wave-front at different depths of our object. There is however one pitfall: the phase is only known modulo 2π (the dashed line in Figure 5B). We show for simplicity only the phase along a line across our phase image. This phase pattern is known as “wrapped” and to get the real depth differences we have to remove the 2π steps, a process called “unwrapping” to obtain the full line in Figure 5B, which is linear related to the real height [42]. In the case of DHM, we have a 2D phase image which has to be unwrapped (Figure 5B). When we have a semi-transparent object we can shine the object beam through the sample (see Figure 5C). Now the phase delay is given by φ(i,j) = 2π δ(i,j)/λ, where the change in optical path length is δ = Δn·t, with Δn(i,j) the change in refractive index and t(i,j) the local thickness of our sample. Δn refers to the difference in refractive index of our sample with the environment (in microscopy n_env_ is 1 in air or the refractive index n_fluid_ of the immersion fluid. With a digital holographic microscope, it is therefore possible to see changes of semi-transparent objects in the vertical direction (change in Δn and/or t), which cannot be detected with a classical microscope. An optical scheme of such a digital holographic microscope is given in Figure 5C. We used in this study a DHM T1000 digital holographic transmission microscope (Figure 5A–I) from Lyncée Tec (Lausanne, Switzerland) [43]. This microscope uses an off-axis configuration with a Mach–Zehnder interferometer and is equipped with a 666 nm laser diode with very low illumination power (200 μW/cm^2^).

### 3.6. Equipment and Settings

The holograms are registered with a CCD camera (1024 × 1024 pixels, 30 fps), using a 20× objective (N.A. = 0.7; FOV = 330 μm; no immersion). The objective lens and the condenser assembly were designed to measure through a 0.17 mm thick cover glass. The lateral resolution was 0.1 µm and the axial below 1 nm. Imaging up to a depth of 300 μm is possible. Data acquisition and evaluation were performed using the Koala Software of Lyncée Tec [44].

### 3.7. Statistical Analysis

The statistical evaluation was performed using the Statistica 13 software. To be able to perform statistical analysis of variances (ANOVA), two conditions: normality of the distribution as well as homogeneity of variances were verified. To assess the normality of distribution the Shapiro–Wilk test was performed, while homogeneity of variances was tested (a) in case of a normal distribution—by Hartley’s test and (b) when the data analyzed did not meet the assumption of distribution normality—by Levene’s and Brown–Forsythe tests. We choose to evaluate the statistical significance with *p* < 0.05. The results (the averages of the control resp. the and test groups) were compared by the one-way analysis of variance (ANOVA) in case these two assumptions were met. A non-parametric counterpart of ANOVA, the Kruskal–Wallis test by ranks, was performed when the assumptions of normality or homogeneity of variances were not met by the data analyzed. A post hoc analysis was carried out to determine more precisely between which groups of results the means differed. The data analyzed by the ANOVA were evaluated using Tukey’s test. After a non-parametric evaluation of the equality of variances, we carried out Dunn’s test.

## 4. Conclusions

The usefulness of digital holographic microscopy in ex vivo human skin examination, including the SC layer, has been proven for the first time (Figure 5A–I). The canyons (intercluster region) surrounding the clusters (corneocyte agglomeration) have been identified as an important pathway for penetration of substances into the skin. This increases our knowledge about substances (molecular weights higher than 500 Da) that are able to overcome the skin barrier by a pathway different from the well-known transcellular, intercellular, and transappendageal ones. The data obtained enabled us for the first time to study the complex organization of the SC as a three-level model, which is more advanced than the ones described in current scientific literature (Table 2) [45,46]. The knowledge we acquired provides the information necessary to understand the mechanisms of overcoming the skin barrier by various substances (e.g., therapeutic or toxic), including uncontrolled diffusion to the *SC*, and then permeation into the epidermis and even into the dermis, where the blood and lymphatic vessels are located. It has been proven that cyclic siloxanes interact with the structures of the *SC* causing irreversible damage by affecting:

(a) the first level of the barrier—destabilization of the lipid bilayer resulting in the destruction of the corneocyte structure, observed as a change in geometry with an axial resolution of nanometers and even collapse in space. We can conclude that these compounds have affinity for amphiphilic structures of the lipid bilayer due to the lipophilic properties of cyclic siloxanes ((logPo/w 5.10—about 9), resulting in a change in their conformation, e.g., orthorhombic (most regular and densely packed), responsible for the largest barrier, and hexagonal (slightly relaxed), to liquid crystal (relaxed conformation responsible for the reduction of the barrier), and even irreversible lipid extraction;
(b) the second level of the barrier—destruction of the structure of the lipid bilayer causing the collapse of not only corneocytes, but also a significant part of the clusters, which leads to the loss of the SC integrity and lacunae formation. The lacunae occurring might cause transepidermal drug delivery or enhanced penetration of undesirable substances. Lipophilic siloxanes can also interact with lipid canyons. Obtaining further knowledge is required.
(c) the third level of the barrier—changing the topography of the SC surface and interrupting the barrier continuity of this skin layer, measured with a lateral resolution of micrometers. On the basis of the results obtained, we found that of the cyclic siloxanes tested, siloxane D6 disturbs the integrity of the SC, and thus reduces the skin barrier less then D5 and especially D4. Additional additional research to increase our knowledge is required.

Studies with focus on multilevel structure in the skin barrier function, stratum corneum disruption due to interaction with the xenobiotics, skin barrier restoration strategies, active substances diffusion to skin, penetration pathways, as well as toxicological studies, could benefit tremendously from the presented technology.

## Figures and Tables

**Table 1:** The results of the stratum corneum geometrical topography and structural changes as a consequence of impact of cyclic siloxanes (*n* = 7).

Imaging	Feature	Sample	1	2	3	4	5	6	7	Mean	SD	RSD (%)
Figure 1I–L Figure 4A	Width of the corneocyte/lacunae (μm)	Control sample	38	30	31	28	35	35	34	33	4	11
		D4	100	130	104	110	104	104	90	106	12	12
		D5	76	104	126	117	100	122	104	107	17	16
		D6	104	100	130	91	117	104	100	107	13	12
Figure 1I–L Figure 4B	Depth alteration in skin topography (μm)	Control sample	54	72	64	48	72	48	48	58	11	19
		D4	140	160	120	104	96	160	92	125	29	23
		D5	194	184	160	120	144	112	102	145	36	25
		D6	133	136	112	157	120	144	136	134	15	11
Figure 2A–D Figure 4C	Phase change in skin topography (°)	Control sample	4149	4443	3977	3310	2927	2397	5464	3810	1025	27
		D4	5477	7610	6272	10,561	7964	7349	8365	7657	1623	21
		D5	5561	4869	5819	4681	9509	7059	6268	6252	1649	26
		D6	5560	6986	6655	6031	4806	4476	6565	5868	961	16
Figure 2E–H Figure 4D	Distance between max./min. phase value (μm)	Control sample	45	47	45	42	48	45	43	45	2	5
		D4	120	100	130	130	120	120	120	120	10	8
		D5	130	140	130	130	110	150	120	130	13	10
		D6	110	80	80	90	80	100	90	90	12	13
Figure 2E–H Figure 4E	Phase change in skin topography—profile line (°)	Control sample	800	1000	1400	1000	800	800	1200	1000	231	24
		D4	1900	1600	1800	2000	3100	1700	2200	2043	506	23
		D5	3000	1100	1100	1400	2000	1700	1600	1700	658	25
		D6	1300	2300	1200	1400	1400	1600	1800	1571	377	24

**Table 2:** Proposal for three-level organization of the stratum corneum barrier [8,27,28,29,31,32,33,45,46,47,48,49].

Level of Organisation	Stratum Corneum Component	Structural Characteristics	Impact of the Skin Barrier Function
First	Corneocyte (contribution—70%)	-a single, dead, flattened cell, with regular shapes, e.g., hexagonal, pentagonal and diameter approx. 10–40 µm; -the building blocks of the internal structure are: (a) stable protein—about 20 varieties of keratin, including α- keratin 58% which is organized in dense filaments, which extend throughout the cell. It causes reinforcement of the skin cells and provide structural support, (b) other proteins—10% (filaggrin, involucrin, loricrin, cornifin, trichonyalin (c) NMF 30%; -Corneocyte surrounded by a protective peripheral envelope: (a) from the inside—the cornified cell envelope (CCE), consisting of crosslinked cytosolic proteins: involucrin, loricrin, keratin, filaggrin, trichonyalin (TTH) and in a small extent small proline-rich proteins, (b) from the outside—corneocyte lipid envelope (CLE), composed of long-chain ω-OH-ceramides and long-chain ω-OH-fatty acids; -There is a strong covalent bond between CCE and CLE (binding energy 300 kJ × mol^−1^), which strengthens the corneal stiffness. The basis is the crosslinking of involucrin with structural proteins, creating a suitable substrate for the combination of CCE and CLE. This connection occurs via a covalent bond described as an ester link, in which a carbonyl group of respectively arrayed glutamic acid residues of involucrin is bound to ω-hydroxy ULC-ceramides and free fatty acids; -The corneocytes are connected by corneodesmosomes	1. the smallest structurally level of skin barrier 2. maintenance of the mechanical stability
	lipid matrix (contribution—20%)	-multilayer structure composed of lipid bilayers—width 12 nm- a thermodynamically stable self-assembly system, maintained by van der Waals bonds, hydrogen and electrostatic bonds; these bilayers form regions of semicrystalline, gel and liquid crystals domains; most molecules penetrate through the skin via this intercellular microroute and therefore many enhancing techniques aim to disrupt or bypass its highly organized structure; -The building block of the structure is a mixture of: (a) ceramides (30–40%)—heterogeneous moieties in which the free fatty acids are connected by an amide bond to the sphingosine base; the acyl chain length in ceramides is mostly C24–C26 what gives rigidity (b) cholesterol and its esters (25%)—filling the intercellular spaces and increasing the cohesion of the layer and the water-tight barrier (c) free fatty acids (18%) (d) cholesteryl sulfate (5%) (e) triglicerydes (f) hydrocarbons (11%), -with the lipoprotein CLE corneocyte envelope is connected with van der Waals bonds (binding energy 2–4 kJ × mol^−1^), -the skin barrier function is determined by: (a) the lamellar organization of the chains of intercellular lipids, which can have three main conformations: the orthorhombic structure—thermodynamically stable, the less permeable and highly ordered states, the hexagonal structure—less thermodynamically stable and more permeable and disordered than orthorhombic and the fluid state (liquid); fluid state—the least thermodynamically stable and the most permeable and disordered; (b) the lateral organization in accordance with the distance between the chains of the intercellular lipids: orthorhombic (0.375–0.41 nm), hexagonal (0.41 nm) and liquid (0.46 nm)	1.guarantee skin barrier (limits permeability of substances, allergens and microorganisms)
Second	Clusters	-specific organization approx. 15–30 corneocytes (that range from 100–250 µm in width across the surface), and 150–300 cells close to the basal layer separated by canyons—intercluster spaces, intercluster region	1. strengthening mechanical stability
	Canyons	-canyons (intercluster region)—the invaginations or microfolds of the stratum corneum cell layers, the intercluster spaces (width ranging from 10–30 µm); -structurally built of lipids; hydrophobic and lipophilic properties; -in the surface the intercluster regions start as small wrinkles and deeper into the skin, these wrinkles close and are replaced by canyons; -a cross-section perpendicular to the skin surface, the canyons appear as invaginations of the SC into the tissue -the canyons can be observed up to 58 μm depth from the surface of the tissue, 6 μm away from the dermis	1. structure can even extend in depth to dermoepidermal junction, which allows xenobiotics to diffuse even directly into blood or lymph vessels, omitting stratum corneum lipids
Third	Compact surface	-skin surface with regular cells -specific and compact structural organization composed of tightly adhering corneocytes, surrounded by an extracellular lipid matrix (lipid—enriched extracellular matrix). -layered construction—15–20 layers with a total thickness of 10–20 μm (thick) -the integrity of the layer is also maintained by the corneodesmosomes—intercellular proteins that combine with the cohesion forces with adjacent corneocytes, both in the plane of a single layer of stratum corneum and with a deeper neighboring layer; directly related to the exfoliation process.	1. maintenance of tightness and flexibility 2. barrier function
